# Aerosoltherapie bei spontan atmenden, tracheotomierten Patienten: eine In-vitro-Studie

**DOI:** 10.1007/s00101-025-01618-2

**Published:** 2025-12-18

**Authors:** Y. Kropp, K. Barthel, G. Beck, M. Thiel, C. Tsagogiorgas, M. Otto

**Affiliations:** 1 Klinik für Anästhesiologie, Operative Intensivmedizin und Schmerzmedizin, Universitätsmedizin Mannheim, Medizinische Fakultät Mannheim, Universität Heidelberg, Theodor-Kutzer-Ufer 1–3, 68167 Mannheim, Deutschland; 2 Klinik für Anästhesie, Intensiv- und Notfallmedizin, St. Elisabethen-Krankenhaus Frankfurt, Frankfurt am Main, Deutschland

**Keywords:** Vibrating-mesh-nebulizer, Tracheostomie, Vernebler, Medikamentendeposition, Weaning, Vibrating-mesh-nebulizer, Tracheostomy, Nebulizer, Drug delivery, Weaning

## Abstract

**Hintergrund:**

Die Aerosoltherapie kommt bei tracheotomierten Patient:innen regelhaft zum Einsatz, z. B. im prolongierten Weaning. Vernebelte Medikamente sollen u. a. die mukoziliäre Clearance verbessern oder eine Bronchoobstruktion behandeln. Es existieren allerdings keine Empfehlungen, wie die Verneblungstherapie bei dieser Patientengruppe am besten durchgeführt werden sollte.

**Ziel der Arbeit:**

Ziel der In-vitro-Studie war es herauszufinden, mit welchem Aerosolgenerator und über welches Interface die Medikamentendepositionsrate bei spontan atmenden, tracheotomierten Patient:innen am höchsten ist.

**Material und Methoden:**

Die Medikamentendepositionsraten eines Jet- (Cirrus™ 2, Intersurgical Beatmungsgeräte GmbH, Sankt Augustin, DE) und eines Vibrating-mesh-Verneblers (Aerogen® Solo, Aerogen Ltd., Galway, IE) wurden in einem In-vitro-Modell spontan und Raumluft atmender, tracheotomierter Patient:innen verglichen. Das Aerosol wurde sowohl über 2 verschiedene Verneblermasken als auch über ein T‑Stück, das direkt an die Trachealkanüle angeschlossen wurde, verabreicht.

**Ergebnisse:**

Der Meshvernebler konnte mittels T‑Stück die höchste Medikamentendepositionsrate erreichen (19,32 ± 4,29 %). Diese war signifikant höher (*p* = 0,008) als die beste Depositionsrate des Jetverneblers, die dieser unter Verwendung einer Gesichtsmaske erreichte (12,33 ± 1,38 %).

**Diskussion:**

Die regelhafte Verwendung von Vibrating-mesh-Verneblern, die zur Medikamentenverneblung mittels T‑Stück direkt an die Trachealkanüle konnektiert werden, könnte die Aerosoltherapie von spontan atmenden, tracheotomierten Patient:innen verbessern.

**Zusatzmaterial online:**

Die Online-Version dieses Artikels (10.1007/s00101-025-01618-2) enthält die mit einem S gekennzeichneten Zusatzelemente. Bitte scannen Sie den QR-Code.

## Abstract (en)

**Background:**

Aerosol therapy is widely used in tracheostomized patients, particularly during prolonged weaning, to improve mucociliary clearance and manage bronchoconstriction; however, there is a lack of standardized guidelines to optimize nebulization therapy in this patient population. Different nebulizer types and interfaces can affect the efficiency of drug deposition, thereby affecting the therapeutic efficacy. Understanding these differences is essential to improve aerosol delivery strategies in clinical practice.

**Objective:**

This in vitro study aimed to evaluate the drug deposition rates of different aerosol generators and interfaces to determine the most effective combination for spontaneously breathing tracheostomized patients. Specifically, we compared the performance of a jet nebulizer and a vibrating mesh nebulizer in combination with different delivery interfaces: a face mask, a tracheostomy mask and a T-piece connected directly to the tracheostomy tube.

**Material and methods:**

The study used an in vitro model simulating a spontaneously breathing tracheostomized patient. Drug deposition rates were measured using a jet nebulizer (Cirrus™ 2, Intersurgical Beatmungsgeräte GmbH, Sankt Augustin, Germany) and a vibrating mesh nebulizer (Aerogen® Solo, Aerogen Ltd., Galway, Ireland). The aerosol was delivered with the help of a face mask, a tracheal mask and a T-piece. The lung deposition rate was quantified gravimetrically by analyzing salbutamol deposition in a filter placed at the simulated airway.

**Results:**

The vibrating mesh nebulizer demonstrated the highest drug deposition rate (19.32 ± 4.29%) when used with a T-piece, significantly outperforming the jet nebulizer, which achieved its highest deposition rate (12.33 ± 1.38%) with a face mask (*p* = 0.008). The mesh nebulizer also achieved a higher deposition rate than the jet nebulizer when a tracheostomy mask was used (*p* = 0.023 with 1 l O_2_/min supplementary flow, *p* < 0.001 with 8 l O_2_/min).

**Conclusion:**

The results suggest that the use of a vibrating mesh nebulizer connected directly to the tracheostomy tube via a T-piece can improve aerosol delivery efficiency in spontaneously breathing tracheostomized patients. The T‑piece functions as a small reservoir when a vibrating mesh nebulizer is used without additional oxygen supply, improving the deposition efficiency. Conversely, jet nebulizers require a continuous oxygen/air flow for operation; this can result in significant drug loss when used with a T-piece due to the continuous release of aerosol through the distal T‑piece opening. These findings are consistent with previous studies highlighting the benefits of mesh nebulizers in mechanically ventilated patients but extend this knowledge to spontaneously breathing tracheostomized individuals. Further clinical research is required to validate these in vitro results and to assess their impact on patient outcomes, secretion clearance and treatment efficacy in real-world settings.

## Hinführung zum Thema

Spontan atmende und tracheotomierte Patient:innen sind ein häufiges Patientengut auf Intensivstationen, z. B. bei prolongiertem Weaning. Viele dieser Patient:innen erhalten eine Therapie mit vernebelten Medikamenten, z. B. im Rahmen einer bronchodilatativen Therapie oder zur Verbesserung der mukoziliären Clearance. Standardisierte Empfehlungen für die optimale Verneblungstherapie bei dieser Patientengruppe fehlen allerdings. Diese Studie vergleicht die Medikamentendeposition von Jet- und Meshverneblern mit unterschiedlichen Interfaces bei diesen Patient:innen im In-vitro-Modell.

## Hintergrund und Fragestellung

Im Rahmen einer schweren respiratorischen Insuffizienz sind die Intubation und invasive Beatmung ein Standardtherapieverfahren auf deutschen Intensivstationen [12]. Die anschließende Entwöhnung vom Beatmungsgerät stellt hierbei eine große Herausforderung dar und kann bei ARDS-Patient:innen 35 % der gesamten Beatmungszeit in Anspruch nehmen [17]. Gelingt das Weaning nicht, kann eine Tracheotomie indiziert sein [26]. Weitere Indikationen neben prolongierter Beatmungsdauer können u. a. Atemwegsverlegungen (z. B. durch Trauma oder Tumorerkrankungen) oder neuromuskuläre Erkrankungen sein [6, 25].

Die klinische Praxis zeigt, dass tracheotomierte Patient:innen auf Intermediate-Care- und Intensivstationen aus unterschiedlicher Indikation Therapien mit vernebelten Medikamenten erhalten. Zur Unterstützung der mukoziliären Clearance werden häufig vernebelte Medikamente wie isotonische Kochsalzlösung oder bronchodilatative Substanzen bei Vorliegen einer Bronchoobstruktion eingesetzt [13, 14, 18]. Trotz der routinemäßig eingesetzten Aerosoltherapie existieren bislang keine standardisierten Empfehlungen zur optimalen Durchführung bei dieser Patientengruppe.

Zur Verneblungstherapie von Intensivpatient:innen stehen verschiedene Aerosolgeneratoren zur Verfügung. Jetvernebler werden hierbei am häufigsten verwendet [15, 22]. Diese benötigen zum Betrieb einen Frischgasfluss [2].

Vibrating-mesh-Vernebler werden klinisch immer öfter eingesetzt. Meshvernebler erzeugen das Aerosol elektrisch durch hochfrequente Schwingungen einer perforierten Membran. Im Vergleich zu Jetverneblern bieten sie einige Vorteile: Sie produzieren ein konstantes Aerosol, das die peripheren Lungenbereiche optimal erreicht, und zeigen eine höhere Lungendepositionsrate des vernebelten Medikaments. Auch haben Meshvernebler eine kürzere Verneblungsdauer und benötigen keinen zusätzlichen Frischgasfluss [2].

Klinische und experimentelle Studien haben eine Überlegenheit der Meshvernebler bei invasiv beatmeten Patient:innen gezeigt, die Datenlage für spontan atmende tracheotomierte Patient:innen ist jedoch begrenzt [1]. Im Vergleich von Spontanatmung, nichtinvasiver und invasiver Beatmung kann bei optimaler Positionierung des Verneblers im Beatmungskreislauf die Depositionsrate bei invasiver Beatmung im Vergleich zu Spontanatmung erhöht sein [3]. Bei kontrollierten Beatmungsformen ist v. a. die Positionierung des Verneblers im Beatmungskreislauf entscheidend, da bei Verwendung von Zwei-Schlauch-Systemen, bei denen der inspiratorische und der exspiratorische Schenkel getrennt voneinander verlaufen, eine beatmungsgerätnahe Positionierung möglich ist und damit der inspiratorische Schenkel als „Spacer“ fungiert [19].

Aktuell existieren keine Daten, mit welchem Verneblertyp und über welches kommerziell verfügbare Interface die Aerosoltherapie von spontan atmenden, tracheotomierten Patient:innen ohne zusätzliche Sauerstofftherapie durchgeführt werden soll.

## Zielsetzung

Ziel dieser In-vitro-Studie war es, die Medikamentendepositionsrate verschiedener Verneblersysteme (Jet- vs. Meshvernebler) in Kombination mit unterschiedlichen Interfaces bei simulierten spontan atmenden, tracheotomierten Patient:innen zu vergleichen. Die Ergebnisse sollen dazu beitragen, die Verneblungstherapie in dieser Patientengruppe zu optimieren und potenzielle klinische Empfehlungen abzuleiten.

## Studiendesign und Untersuchungsmethoden

### In-vitro-Modell spontan atmender, tracheotomierter Patient:innen

**Abb. 1:**
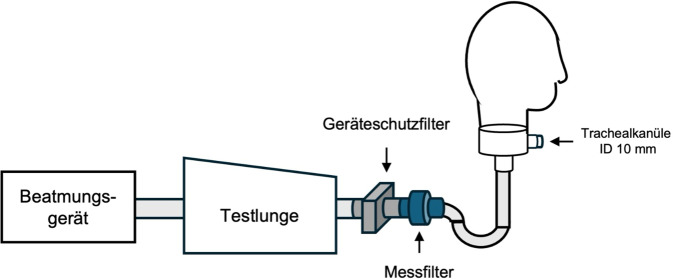
Schematischer Versuchsaufbau

Zur Simulation tracheotomierter Patient:innen verwendeten wir eine Simulationsübungsplatte für Tracheostomiepflege (Fa. Erler-Zimmer, Lauf, DE), die an ein Beatmungsmodell angeschlossen wurde. Über die Tracheostomaöffnung wurde eine #10-Trachealkanüle mit Cuff (Fa. Tracoe medical GmbH, Nieder-Olm, DE) in die dahinterliegende Trachea eingeführt und geblockt. Mithilfe einer Testlunge (Fa. Michigan Instruments, Michigan, US) und eines Beatmungsgerätes (Oxylog 3000 plus, Fa. Dräger Medical Deutschland GmbH, Lübeck, DE) wurde Spontanatmung simuliert (Abb. 1 und 2). Eine detaillierte Beschreibung der Funktionsweise der Testlunge ist im Zusatzmaterial online (Abb. S1) beigefügt.

**Abb. 2:**
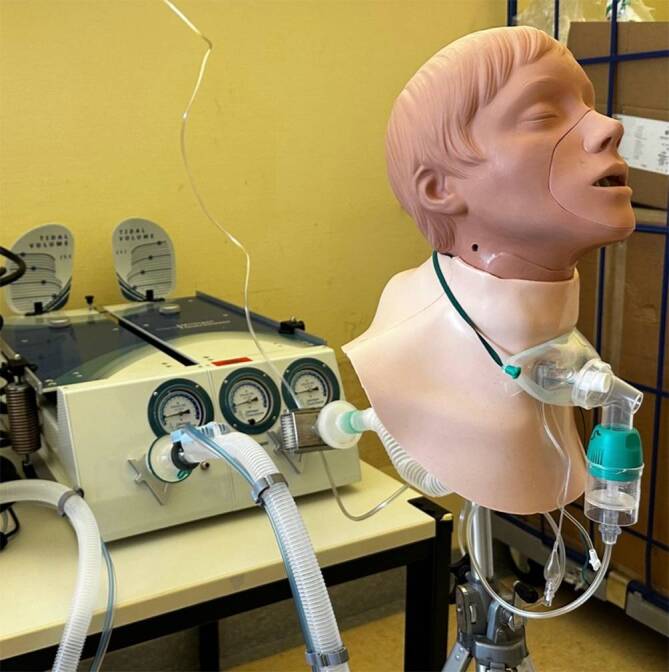
Versuchsaufbau, exemplarisch mit Jetvernebler und Tracheostomamaske

### Atemfrequenz und Tidalvolumen

Um das Spontanatmungsmuster tracheotomierter Patient:innen zu simulieren, wurden analog zum Vorgehen anderer Arbeitsgruppen eine Atemfrequenz von 20 Atemzügen/min, ein Tidalvolumen von 400 ml und ein Inspiration-Exspiration-Verhältnis von 1:2 gewählt [4, 23].

### Vernebler und Interfaces

**Abb. 3:**
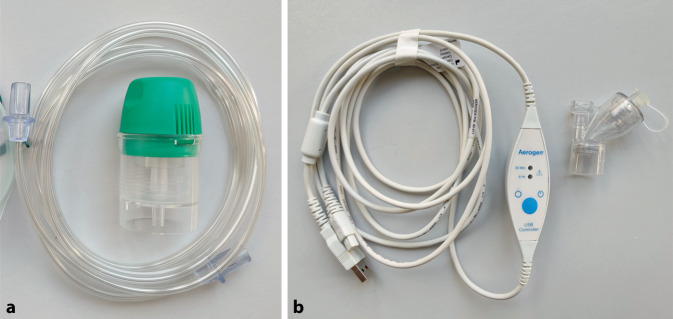
Verwendete Verneblersysteme. **a** Jetvernebler Intersurgical® Cirrus™ 2; **b** Aerogen® Solo

Salbutamol wurde mit 2 Masken und einem T‑Stück unter Verwendung eines Jetverneblers (Cirrus™ 2, Fa. Intersurgical Beatmungsgeräte GmbH, Sankt Augustin, DE) sowie eines Meshverneblers (Aerogen® Solo, Fa. Aerogen Ltd., Galway, IE) vernebelt (Abb. 3).

In den Aufbauten mit Gesichtsmaske wurde jeweils die vom Hersteller mitgelieferte Maske verwendet (Fa. Intersurgical Beatmungsgeräte GmbH, Sankt Augustin, DE und Fa. Aerogen Ltd, Galway, IE). Für die Aufbauten mit Tracheostomamaske wurde für beide Vernebler die Tracheostomamaske der Fa. Intersurgical Beatmungsgeräte GmbH verwendet, da von der Fa. Aerogen Ltd. keine Tracheostomamaske verfügbar ist. Beide Masken wurden so dicht wie im klinischen Setting möglich auf der Simulationsplatte über der Trachealkanüle platziert. Für die Verbindung mittels T‑Stück wurde für beide Vernebler das jeweils vom Hersteller vorgesehene T‑Stück verwendet.

**Abb. 4:**
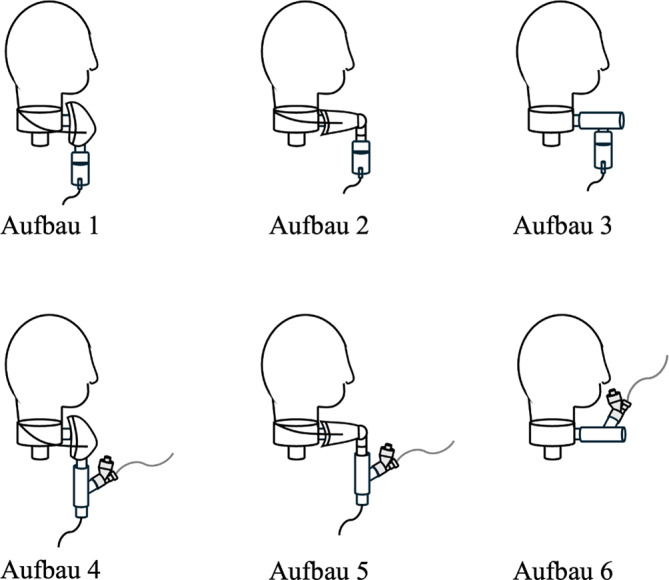
Versuchsaufbauten. *Aufbau* *1*: Jetvernebler + Gesichtsmaske; *Aufbau* *2*: Jetvernebler + Tracheostomamaske; *Aufbau* *3*: Jetvernebler + T-Stück; *Aufbau* *4*: Meshvernebler + Gesichtsmaske; *Aufbau* *5*: Meshvernebler + Tracheostomamaske; *Aufbau* *6*: Meshvernebler + T-Stück

Beim Meshvernebler mit Gesichts- oder Tracheostomamaske war ein Frischgasfluss (Vergleich 1 und 8 l O_2_/min) erforderlich, um das Aerosol ausreichend in Bewegung zu versetzen, sodass es die Strecke bis in die Maske und zur Trachealkanüle zurücklegen kann. Bei Verwendung des T‑Stücks war dies nicht nötig, da die Aerosolabgabe direkt in das T‑Stück erfolgte, sich das Aerosol unmittelbar vor der Trachealkanüle sammeln und dann eingeatmet werden konnte. Der Jetvernebler wurde in allen Versuchsaufbauten mit 8 l O_2_/min betrieben. Die Versuchsaufbauten sind in Abb. 4 dargestellt.

### Studienablauf

Der Ablauf der Versuche erfolgte analog zum Vorgehen vorheriger Arbeiten dieser Arbeitsgruppe mit der von El Taoum et al. vorgeschlagenen gravimetrischen Methode [16]. Aufgrund des zu vernachlässigenden Einflusses von Salbutamol auf die Dichte der Salbutamollösung wurde angenommen, dass 1 ml Salbutamollösung einer Masse von 1 g entspricht [16, 20]. Es wurde eine 2,5-mg/ml-Salbutamollösung (Fa. GSK, London, GB) vernebelt, da diese mithilfe eines Fotometers leicht nachweisbar ist.

Zu Versuchsbeginn wurde die Verneblerkammer mithilfe einer Präzisionswaage (Ablesbarkeit 0,0001 g, Fa. Scaltec Instruments GmbH, Göttingen, DE) gewogen und anschließend mit einer kalibrierten Pipette (Fa. BRAND, Wertheim, DE) mit 2,5 ml der Salbutamollösung befüllt. Die Verneblung wurde daraufhin durchgeführt, bis keine Aerosolbildung mehr sichtbar war. Die Verneblerkammer wurde nach Verneblungsende gewogen und mit destilliertem Wasser ausgewaschen, um mögliche Salbutamolrückstände aus der Verneblerkammer zu lösen.

Der hydrophobe Filter (Respirgard II 303, Fa. Vital Signs Division of CareFusion, San Diego, US) wurde nach dem Verneblungsende vorsichtig aus dem Filtergehäuse gelöst und in ein 50-ml-Probengefäß mit 10 ml destilliertem Wasser verbracht. Das Probengefäß wurde daraufhin 2 min. gevortext. Nach der Hälfte der Zeit wurde der Filter mit einer Pinzette gedreht und gewendet, um ein vollständiges Auswaschen des enthaltenen Salbutamols zu gewährleisten.

Alle Versuche wurden *n* = 5-mal wiederholt.

### Analyse und Quantifizierung des Salbutamols

Die Konzentration des im Eluat enthaltenen Salbutamols wurde fotometrisch bei einer Wellenlänge von 276 nm gemessen [10, 20]. Für jeden Durchlauf wurde die Salbutamolkonzentration im Filtereluat sowie in der Verneblerkammer nach dem Verneblungsende gemessen.

### Leistungsdaten

Gesamtausstoß und Residualvolumen der Vernebler wurden gravimetrisch ermittelt.

### Ermittlung der Lungendeposition

Die *nominale Lungendepositionsrate* ergab sich aus dem Verhältnis der Salbutamolmasse im Filter nach dem Verneblungsende zur vor Versuchsbeginn eingesetzten Salbutamolmasse.

### Statistische Analyse

Bei Vorliegen einer Normalverteilung (*p* > 0,05 im Shapiro-Wilk-Test) wurde bei Varianzgleichheit (Levene-Test > 0,05) ein ungepaarter T‑Test zum Vergleich der Mittelwerte durchgeführt. Bei Ungleichheit der Varianzen (Levene-Test < 0,05) wurde ein Welch-Test durchgeführt. Lag keine Normalverteilung vor, wurde ein Mann-Whitney-U-Test durchgeführt. Verhältnisskalierte Variablen wurden mit Mittelwert ± Standardabweichung berichtet. Die statistische Signifikanz wurde auf *p* < 0,05 festgelegt. Die statistische Analyse erfolgte mit IBM SPSS Statistics 25.

## Ergebnisse

### Interfaces

*Gesichtsmaske*: Bei der Verwendung einer Gesichtsmaske zeigte der Jetvernebler eine Depositionsrate von 12,3 ± 1,4 %. Der Meshvernebler erreichte bei 1 l/min eine Depositionsrate von 11,6 ± 1,4 % und bei 8 l/min 12,8 ± 2,6 %. Die Unterschiede zwischen den beiden Verneblern waren statistisch nicht signifikant.

*Tracheostomamaske*: Mit einer Tracheostomamaske erreichte der Jetvernebler eine Depositionsrate von 9,5 ± 0,9 %, während der Meshvernebler bei 1 l/min 13,6 ± 3,2 % und bei 8 l/min 15,4 ± 0,8 % erzielte. Hier zeigten sich signifikante Unterschiede zugunsten des Meshverneblers.

*T‑Stück*: Beim Einsatz eines T‑Stücks betrug die Depositionsrate des Jetverneblers 5,2 ± 2,2 %, während der Meshvernebler 19,3 ± 4,3 % erreichte. Auch dieser Unterschied war statistisch signifikant.

**Abb. 5:**
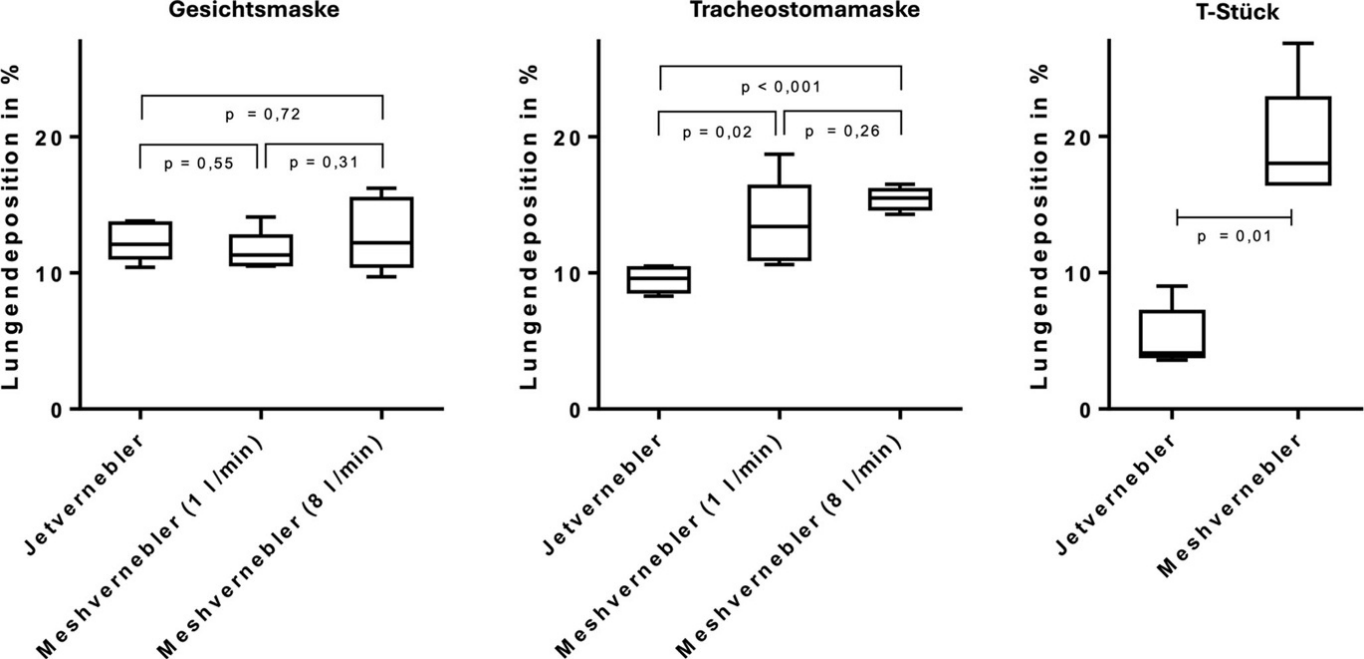
Vergleich der Lungendepositionsraten von Jet- und Meshvernebler mit unterschiedlichen Interfaces. Alle Versuche wurden *n* = 5-mal wiederholt. Boxplot-Darstellung mit Median, Interquartilsabstand und Min./Max

Die Ergebnisse sind auch in Abb. 5 und Tab. 1 dargestellt.

**Tab. 1:** Lungendepositionsrate von Salbutamol der unterschiedlichen Vernebler und Interfaces

	Vernebler		*p*-Wert
Interfaces	Jetvernebler	Meshvernebler	
Gesichtsmaske	12,33 ± 1,38 % (*n* = 5)	11,57 ± 1,43 % (1 l/min) (*n* = 5)	0,548^#^
		12,83 ± 2,60 % (8 l/min) (*n* = 5)	0,715*
Tracheostomamaske	9,50 ± 0,93 % (*n* = 5)	13,64 ± 3,20 % (1 l/min) (*n* = 5)	0,023*
		15,42 ± 0,79 % (8 l/min) (*n* = 5)	< 0,001*
T‑Stück	5,23 ± 2,18 % (*n* = 5)	19,32 ± 4,29 % (*n* = 5)	0,009^#^

Darstellung der Lungendepositionsraten als Mittelwert ± SD; *p*-Werte auf Basis eines ungepaarten T‑Tests (*) oder Mann-Whitney-U-Tests (^#^)

### Verneblersysteme

*Jetvernebler*: Beim Jetvernebler waren die Depositionsraten der Aufbauten mit Gesichtsmaske und Tracheostomamaske im Vergleich zum Aufbau mit T‑Stück signifikant höher (Gesichtsmaske: *p* = 0,008; Tracheostomamaske: *p* = 0,032). Der Aufbau mit Gesichtsmaske zeigte eine signifikant höhere Depositionsrate als der Aufbau mit Tracheostomamaske (*p* = 0,005).

*Meshvernebler: *Bei Verwenden eines Meshverneblers zeigte sich zwischen den Aufbauten der Gesichts- und Tracheostomamaske kein signifikanter Unterschied, unabhängig vom anliegenden Frischgasfluss (bei 1 l O_2_/min: *p* = 0,31; bei 8 l O_2_/min: *p* = 0,09). War der Meshvernebler direkt mittels T‑Stück an die Trachealkanüle konnektiert, war die Depositionsrate signifikant höher als mit Gesichts- oder Tracheostomamaske (*p* = 0,008).

*Vergleich Mesh‑/Jetvernebler:* Im Vergleich der Aufbauten von Mesh-/bzw. Jetvernebler mit den jeweils höchsten Depositionsraten zeigte der Meshvernebler mit T‑Stück (19,3 ± 4,3 %) eine signifikant höhere Depositionsrate als der Jetvernebler mit Gesichtsmaske (12,3 ± 1,4 %), *p* = 0,008.

Die Leistungsdaten der Verneblersysteme (Verneblungsdauer, Gesamtausstoß und Residualvolumen) sind in dem Zusatzmaterial online (Tab. S1) dargestellt.

## Diskussion

Die vorliegende Studie untersucht die Medikamentendeposition bei simulierten spontan atmenden, tracheotomierten Patient:innen unter Verwendung von Jet- und Meshverneblern mit unterschiedlichen kommerziell verfügbaren Interfaces. Die Studienergebnisse konnten zeigen, dass mittels Meshvernebler im In-vitro-Modell die höchste Depositionsrate erzielt werden konnte.

### T-Stück

Der Meshvernebler mit direkter Verbindung an die Trachealkanüle via T‑Stück konnte unter allen Versuchsaufbauten die höchste Depositionsrate erzielen (19,3 %). Diese Ergebnisse decken sich mit denen von Albuainain et al., die eine vergleichbare Depositionsrate (20,2 %) berichteten [1]. Zielsetzung und Aufbau von Albuainain et al. unterschieden sich allerdings durch den Fokus auf die Bewertung des Einflusses unterschiedlicher Sauerstoffflüsse und des Einflusses aktiver Befeuchtung von unserer Studie. Weiterhin wurde die Verneblung ohne zusätzlich anliegenden (Sauerstoff)Fluss mittels T‑Stück und Meshvernebler nicht untersucht.

Im Gegensatz zum Meshvernebler zeigte die Verwendung eines T‑Stücks beim Jetvernebler die niedrigste Depositionsrate. Ältere Studien zeigten hingegen eine bessere Depositionsrate bei Verwendung eines T‑Stücks im Vergleich zu einer Tracheostomamaske [5, 23]. Die Unterschiede könnten durch Unterschiede im In-vitro-Modell erklärbar sein, da in diesen Studien kurze Verbindungsstücke zwischen T‑Stück und Trachealkanüle platziert wurden, was den Reservoireffekt begünstigen könnte. Durch die Verbindungsstücke entsteht zusätzlicher Raum, in dem sich das entstehende Aerosol in der Zeit zwischen Exspiration und Inspiration sammeln und schließlich über das Tracheostoma eingeatmet werden kann. Albuainain et al. verwendeten für das T‑Stück einen mit unserer Studie ähnlichen Aufbau und kamen damit zu vergleichbaren Ergebnissen (7,2 ± 0,5 %) [1].

Jetvernebler benötigen zum Betrieb eine Druckluftquelle mit einem Fluss von – je nach Herstellervorgabe – in der Regel mindestens 6 l/min. Durch diesen Fluss wird das Aerosol bei Verwendung eines T‑Stücks auch in der Exspirations- oder Ruhephase aktiv aus dem distalen Ende des T‑Stücks herausgetrieben, was zum Verlust von Medikament führt. Der Meshvernebler benötigt keine additive Sauerstoff- oder Druckluftzufuhr, weshalb das T‑Stück während der Ruhephase als Reservoir dienen kann und das in dieser Zeit vom Vernebler gebildete Aerosol dort gesammelt wird und dann eingeatmet werden kann.

Die separate Steuerbarkeit von Verneblungs- und Sauerstofftherapie bei Meshverneblern hat weitere Vorteile [9]. Eine Steuerung der F_I_O_2_ ist beim Jetvernebler kaum möglich, da diese im Falle der Verneblung mit Sauerstoff bei Flussraten von 6–15 l O_2_/min im Bereich von 42–47 % liegt [7].

### Gesichts- und Tracheostomamaske

Der Meshvernebler in Kombination mit einer Tracheostomamaske zeigte im Vergleich zum Jetvernebler eine signifikant höhere Depositionsrate. Auch dieses Ergebnis deckt sich, trotz Unterschieden im Versuchsaufbau, mit der Studie von Albuainain et al. [1]. Der gefundene Unterschied lässt sich nicht allein durch den Frischgasfluss des Jetverneblers und den dadurch entstehenden Aerosolverlust durch eine nicht komplett dicht sitzende Maske erklären, da auch der Meshvernebler mit einem niedrigen und einem hohen supplementären Frischgasfluss getestet wurden. Die Unterschiede zwischen den Verneblertypen könnten in unterschiedlichen Leistungsdaten, Residualvolumina oder in Unterschieden im Depositionsverhalten, welches sich u. a. durch unterschiedlich große produzierte Tröpfchen ergibt, begründet liegen.

Interessanterweise zeigten sich bei Verwendung einer über dem Tracheostoma platzierten *Gesichtsmaske* keine signifikanten Unterschiede in der Depositionsrate der beiden Verneblertypen. Dieses Ergebnis entsprach nicht der Hypothese und auch nicht den Ergebnissen der anderen Versuchsaufbauten aus Tracheostomamaske und T‑Stück, bei denen der Meshvernebler eine teils deutliche Überlegenheit zeigte. Einen möglichen Erklärungsansatz stellte ein Unterschied der verwendeten Gesichtsmasken dar; für jeden Verneblertyp wurden die vom jeweiligen Hersteller mitgelieferten Gesichtsmasken verwendet. Die Gesichtsmaske des Jetverneblers Cirrus™ 2 hat eine Gummilippe, die sich an den Hals der Patient:innen (bzw. dieses Tracheostomamodells) anlegt und diesen besser abdichtet. Die Gesichtsmaske des Meshverneblers Aerogen® Solo hat keine Gummilippe, aber dafür 2 Ventile, die den Aerosolverlust in der Atemruhelage verringern sollen (Zusatzmaterial online: Abb. S2). Da seitens Aerogen® keine spezielle Tracheostomamaske verfügbar war, wurden die Versuchsreihen beider Vernebler mit der Tracheostomamaske des Cirrus™ 2 durchgeführt, die ebenfalls eine Gummilippe besitzt.

Zur Überprüfung dieser Hypothese wurden die Vernebler an die Gesichtsmaske des jeweils anderen Herstellers angeschlossen. Bei der Kombination von Jetverneblermaske und Meshvernebler zeigte sich nun tatsächlich eine signifikant höhere Depositionsrate (Meshvernebler (8 l/min): 15,28 ± 1,44 %; Jetvernebler: 12,33 ± 1,38 %; *p* = 0,011). Bei der Gesichtsmaske von Aerogen® ohne abdichtende Gummilippe unterschieden sich Jet- und Meshvernebler nicht signifikant. Die Ergebnisse sind ausführlich im Zusatzmaterial online ( Tab. S2) dargestellt.

Diese Ergebnisse zeigen klar den Einfluss der verwendeten Maske, bei der es auf einen möglichst dichten und passenden Sitz am Hals der Patien:innen ankommt, sodass der Reservoireffekt maximal ausgeschöpft werden kann. Es ist somit auch zu erwarten, dass verschiedene Patient:innen von der Verwendung unterschiedlicher Masken profitieren könnten. Vergleichbare Studien, in denen die Depositionsrate einer auf dem Tracheostoma aufliegenden Gesichtsmaske gemessen und verglichen wurde, existieren nicht.

In vivo spielt neben der Gesamtdepositionsrate die regionale Verteilung des Aerosols in der Lungenperipherie für die klinische Wirksamkeit des vernebelten Medikaments eine große Rolle. Die Deposition in den unteren Atemwegen wird dabei durch die Partikelgröße des produzierten Aerosols beeinflusst, wobei ein „Mass Median Diameter“ (MMD) von 1–5 µm als ideal betrachtet wird [8]. In einer anderen Arbeit dieser Arbeitsgruppe konnte gezeigt werden, dass sich die MMD beider Vernebler nicht signifikant unterscheiden und die hier gefundenen Unterschiede nicht auf eine unterschiedliche Tröpfchengröße zurückgeführt werden können [21].

Zusammengefasst demonstrieren die hier vorliegenden In-vitro-Daten eindrücklich, dass die Depositionsraten des Meshverneblers unabhängig vom verwendeten Interface deutlich höher waren als die Raten eines Jetverneblers. Im direkten Vergleich der Versuchsaufbauten von Jet- und Meshvernebler mit den jeweils höchsten Depositionsraten (Jet: Gesichtsmaske vs. Mesh: T‑Stück) zeigte der Jetvernebler eine um 56 % geringere Depositionsrate. Auch die Güte der verwendeten Verneblermaske scheint signifikanten Einfluss auf diese Ergebnisse zu haben.

## Limitationen

Erkenntnisse aus der Medikamentendeposition in vitro sind nur eingeschränkt auf die Deposition in vivo übertragbar. In den In-vitro-Versuchen wurde auf eine optimale Verneblerposition geachtet, da kleine Positionsänderungen die Verneblung v. a. beim Jetvernebler stark beeinflussen können. In der Weaning-Phase ist eine optimale Positionierung des Verneblers (beim Jetvernebler möglichst 90° mit horizontalem Medikamentenspiegel in der Verneblerkammer) häufig nicht möglich. Eine weitere Verringerung der Depositionsrate durch eine Schräglage des Oberkörpers (z. B. 30–45°) ist erwartbar. Bei Verwendung von Masken hängt die Depositionsrate weiterhin von der optimalen Position der Maske ab. Die Depositionseigenschaften und damit die klinische Wirksamkeit vernebelter Medikamente hängt ferner nicht nur von der totalen Lungendeposition, sondern auch von der regionalen Verteilung der Aerosolpartikel innerhalb der Atemwege ab; Letztere wurde in diesem In-vitro-Modell nicht simuliert [24]. Weitere, bauartbedingte Limitationen des Modells werden im Zusatzmaterial online erläutert. Zuletzt ist eine Unterschätzung der Depositionsrate von Salbutamol denkbar, da der verwendete Respirgard-II-Filter kein „absolut“ hydrophober Filter ist [11].

## Fazit für die Praxis

- Bei der Verneblungstherapie von spontan atmenden, tracheotomierten Patient:innen ohne zusätzliche Sauerstofftherapie konnte ein Meshvernebler, der mittels T‑Stück an die Trachealkanüle konnektiert war, die höchste Medikamentendepositionsrate erreichen.
- Diese war signifikant höher als die beste Depositionsrate des Jetverneblers, die dieser unter Verwendung einer Gesichtsmaske erreichte.
- Klinische Studien müssen aber zeigen, ob die Verwendung von Meshverneblern das Outcome von spontan atmenden, tracheotomierten Patient:innen optimieren kann.

## Supplementary Information

Zusatzmaterial

## Data Availability

Die während dieser Studie generierten und analysierten Daten sind nicht öffentlich zugänglich, können jedoch auf begründete Anfrage vom korrespondierenden Autor bereitgestellt werden.
