# Supplementary material for: Aerosol therapy in spontaneously breathing tracheostomized patients: an in vitro study
Source: Anaesthesiologie. 2025 Dec 18;75(1):12–9. [Article in German] doi: 10.1007/s00101-025-01618-2 (PMC12804279; doi:10.1007/s00101-025-01618-2)
Supplement: Supplementary file 1 — Zusatzmaterial [file 101_2025_1618_MOESM1_ESM.pdf]

Zusatzmaterial zum Beitrag „**Aerosoltherapie bei spontan atmenden, tracheotomierten Patienten: eine *in vitro* Studie**" von Kropp Y, Barthel K, Beck G et al. (2025) in *Die Anaesthesiologie.*

Beitrag und Zusatzmaterial stehen Ihnen auf www.springermedizin.de zur Verfügung. Bitte geben Sie dort den Beitragstitel in die Suche ein.

Supplement zu: Aerosoltherapie bei spontan atmenden, tracheotomierten Patienten: eine *in vitro* Studie

Kropp Y[1], Barthel K[1], Beck G[1], Thiel M[1], Tsagogiorgas C[1,2]*, Otto M[1]

[1] Klinik für Anästhesiologie Operative Intensivmedizin und Schmerzmedizin, Universitätsmedizin Mannheim, Medizinische Fakultät Mannheim, Universität Heidelberg

[2] Klinik für Anästhesie und Intensivmedizin, St. Elisabethenkrankenhaus Frankfurt, Frankfurt am Main

Korrespondenz:
Prof. Dr. med. Charalambos Tsagogiorgas
Klinik für Anästhesiologie, Operative Intensivmedizin und Schmerzmedizin
Universitätsmedizin Mannheim
Medizinische Fakultät Mannheim, Universität Heidelberg
Theodor-Kutzer-Ufer 1-3, 68167 Mannheim
Phone: 0049 (0) 621 383-2415
Fax: 0049 (0) 621 383-2164
Email: charalambos.tsagogiorgas@medma.uni-heidelberg.de

Literatur

1. Alhamad BR, Fink JB, Harwood RJ et al. (2015) Effect of Aerosol Devices and Administration Techniques on Drug Delivery in a Simulated Spontaneously Breathing Pediatric Tracheostomy Model. Respir Care 60:1026-1032
2. Ari A, Fink JB (2016) Differential Medical Aerosol Device and Interface Selection in Patients during Spontaneous, Conventional Mechanical and Noninvasive Ventilation. J Aerosol Med Pulm Drug Deliv 29:95-106
3. Byron PR, Hindle M, Lange CF et al. (2010) In vivo-in vitro correlations: predicting pulmonary drug deposition from pharmaceutical aerosols. J Aerosol Med Pulm Drug Deliv 23 Suppl 2:S59-69
4. El Taoum KK, Xi J, Kim J et al. (2015) In Vitro Evaluation of Aerosols Delivered via the Nasal Route. Respir Care 60:1015-1025
5. Newman SP (1998) How well do in vitro particle size measurements predict drug delivery in vivo? Journal of aerosol medicine : the official journal of the International Society for Aerosols in Medicine 11 Suppl 1:S97-104

S1 Leistungsdaten der verwendeten Verneblersysteme

Verneblungsdauer: Der Jetvernebler benötigte durchschnittlich 409 ± 55 Sekunden, während der Meshvernebler eine durchschnittliche Verneblungsdauer von 382 ± 54 Sekunden aufwies. Der Unterschied war statistisch nicht signifikant (p = 0,13).

Gesamtausstoß: Der Meshvernebler zeigte einen signifikant höheren Gesamtausstoß von 2,38 ± 0,05 ml im Vergleich zum Jetvernebler, der bei gleicher eingesetzter Verneblungslösung 1,78 ± 0,15 ml abgab (p < 0,001).

Residualvolumen: Das Residualvolumen war beim Meshvernebler mit absolut 0,12 ± 0,05 ml geringer als beim Jetvernebler, der nach Verneblungsende 0,72 ± 0,15 ml aufwies. Auch dieser Unterschied war statistisch signifikant (p < 0,001). In Relation zur eingesetzten Flüssigkeitsmenge (2,5 ml) betrug das Residualvolumen beim Jetvernebler 28,8 %, beim Meshvernebler 4,8 %. Die Ergebnisse sind in Tab. S1 zusammengefasst.

Tab. S1: Verneblungsdauer, Gesamtausstoß und Residualvolumen im Vergleich von Jet- und Meshvernebler

	**Jetvernebler**<br>Intersurgical® Cirrus™ 2<br>(n = 15)	**Meshvernebler**<br>Aerogen® Solo<br>(n = 25)	T-Test<br>/ Welch-Test
Verneblungsdauer	409 ± 55 s	382 ± 54 s	P = 0,13
Gesamtausstoß	1,78 ± 0,15 ml	2,38 ± 0,05 ml	P < 0,001
Residualvolumen	0,72 ± 0,15 ml	0,12 ± 0,05 ml	P < 0,001

S2 In vitro Modell eines spontan atmenden, tracheotomierten Patienten

Der Versuchsaufbau ist in Abb. 6 dargestellt. Um Spontanatmung zu simulieren, können beide Testlungen mechanisch miteinander gekoppelt (MK) werden. Nach Kopplung wird die linke Testlunge (TL) nun mithilfe eines Beatmungsgerätes (Oxylog 3000 plus) mit den gewünschten Einstellungen (im vorliegenden Fall mit einer Frequenz von 20/min, einem Volumen von 400 ml und einem I:E von 1:2) überdruckbeatmet. Durch die direkte mechanische Kopplung der beiden Lungen wird nun durch die aktive Exkursion der linken Testlunge die rechte Testlunge (TR) passiv angehoben. Hierdurch wird (reziprok zum Überdruck in der linken Lunge) ein Unterdruck erzeugt, der die simulierte Spontanatmung ausmacht.

Abb. S1: detaillierter Versuchsaufbau

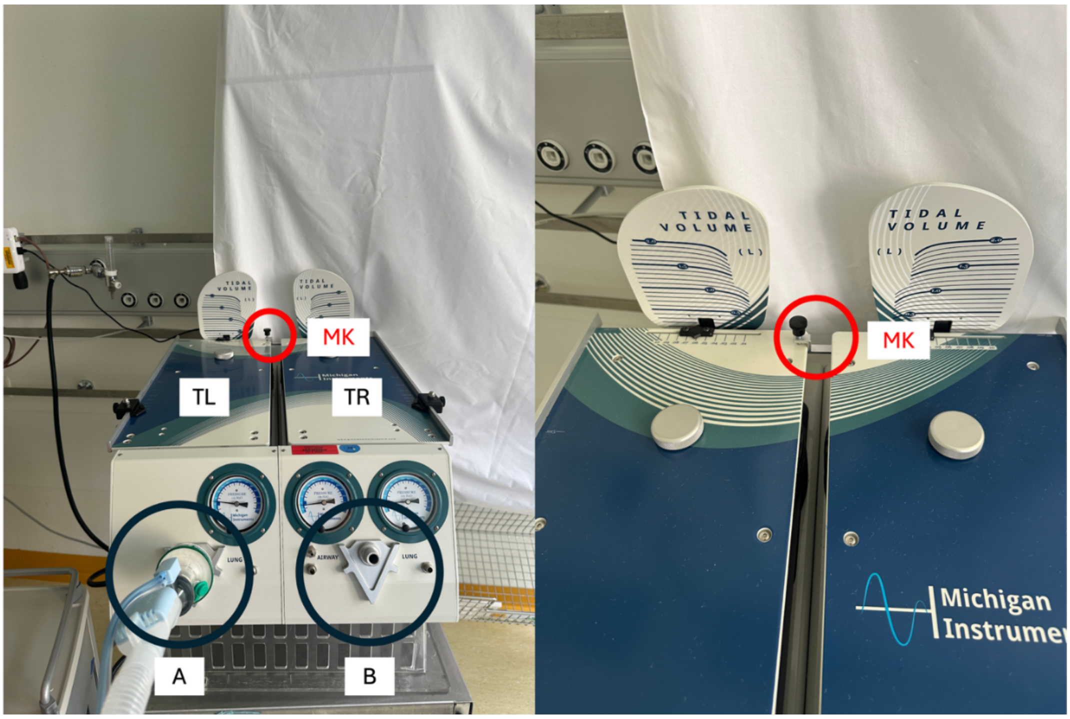

TL: Testlunge links; TR: Testlunge rechts; MK: mechanische Kopplung; A: Verbindung des Beatmungsgerätes (Überdruck); B: Verbindung des Testaufbaus (Unterdruck)

S3 Weitere Limitationen des in vitro Modells

Der Inspirationsvorgang dieses mechanischen Modells kann den tatsächlichen, natürlichen Vorgang nur näherungsweise simulieren, da echte Thoraxmuskulatur- und Diaphragmaaktivität fehlen. Weiterhin bestehen interindividuell und je nach zugrunde liegenden Vorerkrankungen unterschiedliche Eigenschaften von Thorax und Lunge wie Compliance/Elastance, Resistance, Atelektasen und die Temperatur und Feuchtigkeit der Atemwege, die die Aerosoldeposition und -verteilung in den Atemwegen beeinflussen können.

Mit diesem in vitro Modell wird nur die Gesamtdeposition in der Lunge auf Trachealebene simuliert. Dieses Vorgehen entspricht dem etablierten Vorgehen anderer Arbeitsgruppen [1, 2, 4]. Rückschlüsse auf die Aerosolverteilung in der Lungenperipherie können aus dieser Studie daher nicht gezogen werden. Dementsprechend konnten Studien zeigen, dass die in vitro Deposition von Aerosolen im Vergleich zu in vivo Studien überschätzt wird [3, 5].

S4 Einfluss der verwendeten Gesichtsmaske auf die Deposition

Bei Verwendung der jeweils mitgelieferten Gesichtsmaske zeigte sich – im Gegensatz zu den Versuchsreihen mit Tracheostomamaske und mit T-Stück – kein signifikanter Unterschied zwischen Jet- und Meshvernebler. Als Erklärungsansatz kamen Unterschiede zwischen den verwendeten Masken in Betracht, da die Maske des Jetverneblers Cirrus™ 2 im Gegensatz zu der des Meshverneblers Aerogen® Solo eine abdichtende Gummilippe hat, die den Reservoireffekt begünstigen könnte. Aus diesem Grund wurden die Masken in einer anschließenden Versuchsreihe getauscht und beide Vernebler mit der Maske des jeweils anderen Modells getestet. Die Masken sind – exemplarisch adaptiert an den Jetvernebler – in Abb. S2 dargestellt.

In der Versuchsreihe zeigte sich, dass bei Verwendung der Maske des Jetverneblers Cirrus™ 2 mit Gummilippe und in beiden Fällen der Anlage von 8 L/min Sauerstofffluss der Meshvernebler eine signifikant höhere Depositionsrate aufwies (Jetvernebler: 12,33 ± 1,38 %; Meshvernebler (8 L/min): 15,28 ± 1,44 %; p = 0,011). Bei Verwendung der Maske des Meshverneblers Aerogen® Solo zeigten beide Vernebler eine niedrigere Depositionsrate als mit der Maske des Jetverneblers. Der Unterschied in der Depositionsrate zwischen den beiden Verneblern war unter Verwendung der Jetverneblermaske nicht signifikant (Jetvernebler: 11,03 ± 0,87 %; Meshvernebler (8 L/min): 12,83 ± 2,60 %; p = 0,202). Die Ergebnisse sind in der folgenden Tab. S2 dargestellt.

Tab. S2: Vergleich der Depositionsraten der beiden Vernebler unter Verwendung unterschiedlicher Gesichtsmasken

	**Vernebler**		**p-Wert**
**Interfaces**	Jetvernebler	Meshvernebler	
Gesichtsmaske des Jetverneblers (Intersurgical® Cirrus™ 2)	12,33 ± 1,38 % (n = 5)	14,70 ± 1,76 % (1 L/min) (n = 5)<br>15,28 ± 1,44 % (8 L/min) (n = 5)	0,056#<br>0,011*
Gesichtsmaske des Meshverneblers (Aerogen® Solo)	11,03 ± 0,87 % (n = 5)	11,57 ± 1,43 % (1 L/min) (n = 5)<br>12,83 ± 2,60 % (8 L/min) (n = 5)	0,056#<br>0,202+

Darstellung der Lungendepositionsraten als Mittelwert ± SD; p-Werte auf Basis eines ungepaarten T-Tests (*), Welch-Tests (+) oder Mann-Whitney-U-Tests (#)

Abb. S2: Jetvernebler Intersurgical® Cirrus™ 2 mit der mitgelieferten Maske (a) und der Maske des Meshverneblers Aerogen® Solo (b)

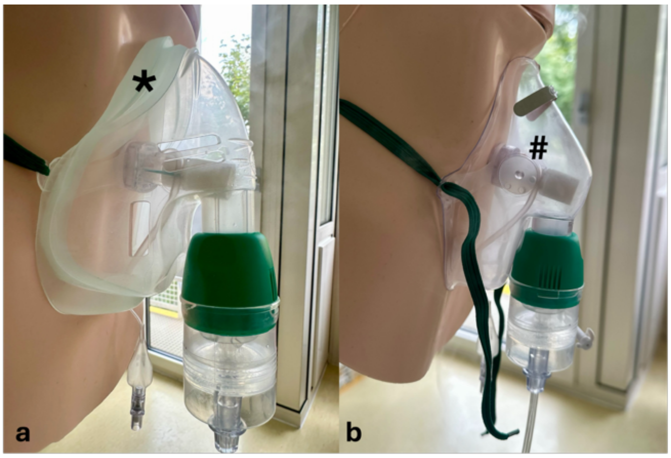

* Gummilippe; # Ventil
